# Assessment of network perturbation amplitudes by applying high-throughput data to causal biological networks

**DOI:** 10.1186/1752-0509-6-54

**Published:** 2012-05-31

**Authors:** Florian Martin, Ty M Thomson, Alain Sewer, David A Drubin, Carole Mathis, Dirk Weisensee, Dexter Pratt, Julia Hoeng, Manuel C Peitsch

**Affiliations:** 1 Philip Morris International R&D, Philip Morris Products S.A., Quai Jeanrenaud 5, Neuchâtel, 2000, Switzerland; 2 Philip Morris International R&D, Philip Morris Research Laboratories GmbH, Fuggerstr.3, Cologne, 51149, Germany; 3 Selventa, One Alewife Center, Cambridge, MA, 02140, USA

## Abstract

**Background:**

High-throughput measurement technologies produce data sets that have the potential to elucidate the biological impact of disease, drug treatment, and environmental agents on humans. The scientific community faces an ongoing challenge in the analysis of these rich data sources to more accurately characterize biological processes that have been perturbed at the mechanistic level. Here, a new approach is built on previous methodologies in which high-throughput data was interpreted using prior biological knowledge of cause and effect relationships. These relationships are structured into network models that describe specific biological processes, such as inflammatory signaling or cell cycle progression. This enables quantitative assessment of network perturbation in response to a given stimulus.

**Results:**

Four complementary methods were devised to quantify treatment-induced activity changes in processes described by network models. In addition, companion statistics were developed to qualify significance and specificity of the results. This approach is called Network Perturbation Amplitude (NPA) scoring because the amplitudes of treatment-induced perturbations are computed for biological network models. The NPA methods were tested on two transcriptomic data sets: normal human bronchial epithelial (NHBE) cells treated with the pro-inflammatory signaling mediator TNFα, and HCT116 colon cancer cells treated with the CDK cell cycle inhibitor R547. Each data set was scored against network models representing different aspects of inflammatory signaling and cell cycle progression, and these scores were compared with independent measures of pathway activity in NHBE cells to verify the approach. The NPA scoring method successfully quantified the amplitude of TNFα-induced perturbation for each network model when compared against NF-κB nuclear localization and cell number. In addition, the degree and specificity to which CDK-inhibition affected cell cycle and inflammatory signaling were meaningfully determined.

**Conclusions:**

The NPA scoring method leverages high-throughput measurements and a priori literature-derived knowledge in the form of network models to characterize the activity change for a broad collection of biological processes at high-resolution. Applications of this framework include comparative assessment of the biological impact caused by environmental factors, toxic substances, or drug treatments.

## Background

Acquisition of large-scale data sets representing a variety of data modalities has become a crucial aspect of experimental system characterization. This strategy enables the broad capture of biological information in a short time and with a relative small investment of effort, in the hope that valuable biological insights might be gained. However, the amount of information collected can be overwhelming, making interpretation of the data difficult and subsequent detailed biological understanding elusive. Reducing the complexity of such data by evaluating it in a relevant biological context is required in order to gain meaningful insight.

**Figure 1:**
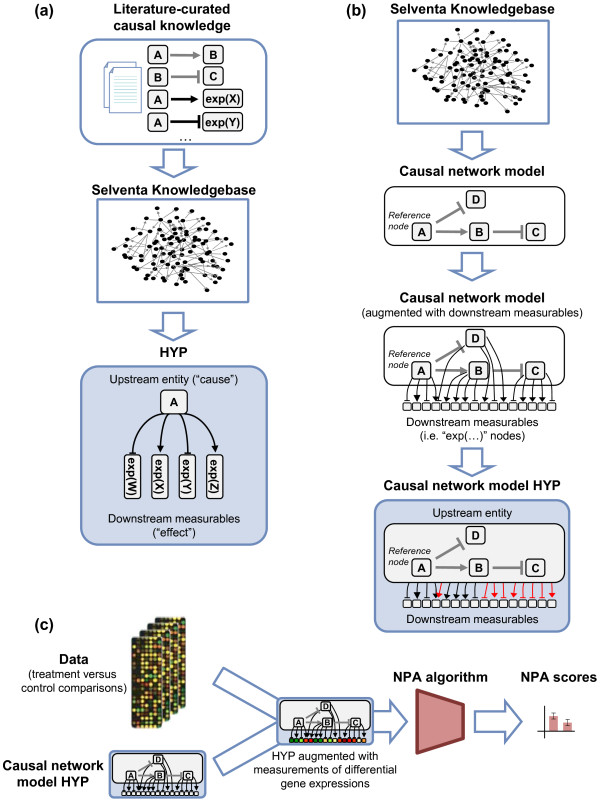
**Constructing and scoring causal network models. (a)** The Selventa Knowledgebase is composed of individual causal relationships that are curated from available sources (generally published scientific literature). For example, in the statement “A → exp(X)” entity A may represent the activity of a particular kinase that has been shown to lead to increased expression of gene X. A HYP is a set of causal relationships derived from the Selventa Knowledgebase that relate an upstream entity to the downstream measurable entities that it regulates. In the HYP example entity A regulates the expressions of genes W, X, Y, and Z following the specific regulation signs “→” (positive regulation) and “--|” (negative regulation). **(b)** An aggregated HYP can be generated from a causal network model, which is a collection of causally linked entities derived from the Selventa Knowledgebase (grey edges). This causal network model can be augmented with the causal relationships to the downstream measureable entities of its nodes (black edges), also derived from the Selventa Knowledgebase. As a consequence, a causal network model can be equivalently viewed as a collection of causally linked HYPs. Next, all the downstream measurable entities of the network model nodes are combined by adjusting their signs based on the causal relationships in the network. This essential step toward the construction of the aggregated HYP is well-defined as long as the network model is causally consistent (see Methods). Because these nodes have a negative causal relationship with the reference node (node A), the regulation signs “→” or “--|” of the downstream measureable entities from nodes C and D are inverted (red edges) when constructing the aggregated HYP from the causal network model. **(c)** NPA scores are calculated from high-throughput data, such as differential gene expression data obtained from treatment versus control comparisons, and applied to an NPA scoring algorithm in the context of a specific causal network model represented by its aggregated HYP.

High-throughput measurements can be evaluated against literature-curated “cause and effect” relationships extracted from the Selventa Knowledgebase (see Methods). As illustrated in Figure 1a, a structure called a “HYP” (derived from “hypothesis”) is used. A HYP is a specific type of network model comprised of a set of causal relationships connecting a particular biological activity (e.g., the increase in abundance or activation of a particular kinase, or a more complex network model describing a growth factor signaling pathway) to measurable downstream entities (e.g., gene expression values) that it positively or negatively regulates. Reverse-causal reasoning (RCR) uses the measurable downstream entities of a HYP to deduce information about the activity of the upstream entity of the HYP, based on an appropriate set of measurements (e.g., differential gene expression from a treatment versus control experiment) [1]. Using measured downstream effects to deduce the activity of upstream entities is advantageous in that, for gene expression data, it does not depend on the “forward” assumption that mRNA expression changes are always directly correlated with protein activity changes [2-4], an assumption that does not take into account the effects of translational or post-translational regulation on protein activity.

The HYP can describe causal relationships between an upstream biological activity and any type of high-throughput data. However, the work described here focuses on the evaluation of whole-genome mRNA expression changes; thus, the HYP is the equivalent of a causal “gene expression signature” for a given entity or process, for example, the activity of a particular kinase. Previous work has explored the importance of uncovering a characteristic signature of gene expression changes that results from one or more perturbations to a biological process, and the subsequent scoring of that signature’s presence in additional data sets as a measure of that process’s specific activity amplitude. Most work in this field has involved identifying and scoring signatures that are correlated with a disease phenotype [5-8]. These phenotype-derived signatures provide significant classification power, but often lack a mechanistic or causal relationship between a single specific perturbation and the signature. Consequently, these signatures may represent multiple distinct unknown perturbations that lead to, or result from, the same disease phenotype.

Alternatively, a number of studies have focused on measuring causal signatures based on very specific upstream perturbations, either performed directly in the system of interest [9] or coming from closely-related published data [10]. Based on the simple, yet powerful premise that modulation of cellular pathways and the components therein is associated with distinct signatures in downstream measurable entities, causally-derived signatures hypothesize that the “cause” of the signature can be identified with high specificity from the measured “effect” [11]. These studies have demonstrated the great potential of applying a causal-pathway activity scoring strategy to clinical problems. For example, they have provided prognosis predictions in gastric cancer patients and indications of specific drug efficacy [10].

As a consequence, coupling specific causal HYPs captured in the Selventa Knowledgebase with a measure of perturbed activity would be a means to further realize this clinical potential, as well as the potential to increase basic biological understanding that is harbored within high-throughput data. However, the HYP infrastructure has been previously exclusively employed as an exploratory tool for identifying relevant perturbed biology by drawing qualitative mechanistic inferences based on statistical enrichments [12-14]. Therefore, new methods are required to confer an explicit, more quantifiable estimate of the degree of HYP activity for a more quantitative comparative assessment infrastructure. Such methods scoring network perturbation amplitudes (NPA) would facilitate a high-resolution comparison of biological states, both by virtue of a continuous scale of scores and the breadth of HYPs that are immediately available for scoring.

To assess HYP amplitude, four complementary scoring algorithms were developed: Strength, Geometric Perturbation Index (GPI), Measured Abundance Signal Score (MASS), and Expected Perturbation Index (EPI). NPA scoring was then applied to different inflammatory and cell cycle-related HYPs using two transcriptomic data sets: a TNFα dose and time series in normal human bronchial epithelial (NHBE) cells and a CDK inhibitor R547 dose and time series in HCT116 colon cancer cells [15]. This study establishes the use of a broad, literature-derived knowledgebase to score the amplitude of various aspects of biology, which can be defined as very specific mechanisms (such as an individual protein activity) that are directly proximal to the data, or as a larger network of interest that is composed of a collection of individual mechanisms.

## Results

### The HYP is the foundation for scoring network models

The HYP represents the relationships between a set of measured data, here gene expression data, and a biological entity that is a known controller of those genes. Additionally, these relationships include the sign (positive or negative) of influence between the upstream entity and the differential expression of the downstream genes. The downstream genes of a HYP are drawn from a database of literature-curated causal biological knowledge (Figure 1a). The causal relationships of a HYP that link the upstream entity to downstream genes are the substrate for the calculation of process amplitude using the NPA scoring algorithms (see below).

A more general causal network model can be constructed from a set of HYPs that are themselves causally connected by literature-derived edges (see Methods). Such a network model can be thought of as providing higher-level connections between HYPs by linking the upstream controllers of these HYPs, based on the pathway’s graph structure. Complex biological processes such as cell proliferation or cellular stress can be efficiently described by causal network models [16,17].

A complex causal network model of biological entities can be transformed into a single HYP by collecting the individual HYPs representing entities in the model and regrouping the connections of all the downstream genes to a single upstream process representing the whole complex causal network model; this in essence is a flattening of the underlying graph structure (Figure 1b). In this fashion, the activity changes of the biological entities described by the network model can be assessed via the aggregation of its individual HYPs, such that the underlying gene expression measurements contribute to the network as a whole (see Methods for a detailed description of how the resulting aggregated HYP is constructed).

### Scoring HYPs with four NPA methods

NPA scoring applies a defined algorithm to an experimental data set consisting in a series of treatment versus control comparisons, where the experimental data is filtered down to a particular scope of biology (and thus a particular set of gene expression relationships) by the context of a defined biological network model (Figure 1c). Specifically, a series of NPA metrics were developed to evaluate the activity of the biological entities represented by a HYP. The NPA metrics were designed such that positive values mean increased activity of the biological entities represented by the HYP (compared to control), and negative values mean decreased activity (compared to control). Furthermore a positive or negative relative difference between two NPA scores denotes the same relative difference in the magnitude of the activity of the biological entities represented by the HYP.

In this study, gene expression data was used to demonstrate the NPA approach using four different scoring methods: Strength, GPI, MASS, and EPI (see Methods). Briefly, Strength is the mean treatment-induced differential expressions of the HYP’s downstream genes, adjusted for the sign of their causal connection to the upstream entity of the HYP. GPI is similar to Strength, except that the contribution of each gene is additionally weighted by taking into account the statistical significance of its differential expression. MASS is the change in absolute downstream quantities in a direction supporting an increase in the upstream entity (i.e., the sum of the measurements corrected for their causal connection to the upstream entity), divided by an average of a total absolute quantity of the downstreams. Finally, EPI is a “smoothed” version of GPI, obtained by averaging a slightly modified form of GPI over all possible values of a threshold for statistically significant differential gene expressions. Each method has specific yet complementary advantages (Table 1), having been tailored for a particular measurement technology or having different conceptual assumptions applied (see Methods). Further complementary aspects of these methods will be discussed below (see Discussion).

**Table 1:** NPA Method Characteristics

**Method**	**Main features**	**Assumptions**	**Pros**	**Cons**
Strength	Linear, unbiased. Based on log_2_ differential measurements (e.g., log_2_ fold changes of measurements).	The contributions from the noisy downstream measurables sum up to zero.	Intuitive.	Noisy/biased signals can artificially decrease/increase the results.
GPI	Based on log_2_ differential measurements. Down-weights weak differential measurements using false non-discovery rates.	The noisy downstream measurables have low false non-discovery rates which can be used to minimize their contributions.	Intuitive. False non-discovery rate depends on the number of experimental replicates.	False non-discovery rate depends on the number of experimental replicates.
MASS	Linear and unbiased in absolute non-log_2_ scale. Dependent on absolute changes in measurements.	Absolute changes in measurements are more important than relative changes.	Intuitive.	Measurements must be directly comparable across all downstream measurables.
EPI	Based on log_2_ differential measurements. Up-weights strong differential measurements without using false non-discovery rates.	The downstream measurables with higher differential values should have stronger contributions than those with lower differential values.	More robust to noisy signals than Strength. Highest sensitivity to strong differential measurements.	Less intuitive. Bootstrapping is needed for calculating Uncertainty.

### Statistical annotation of NPA scores

Each NPA score, regardless of algorithm, represents an abstracted view of a set of biological measurements in the context of a particular HYP. As such, dozens, hundreds, or even thousands of measurements may be aggregated into a single score. In order to better characterize an NPA score and derive value from its use, additional statistics that qualify the score are required. Two such statistics, Uncertainty and Specificity, were developed. Uncertainty is a confidence interval for a particular NPA score, while Specificity tests whether an NPA score is specific to the downstream genes represented by a particular HYP, and not due to a general trend of the data (see Methods).

### NPA scoring of an NF-κB HYP accurately assesses NF-κB activity

In order to evaluate the NPA approach, an NF-κB HYP was scored for a well-understood and controlled experimental system – TNFα-treated NHBE cells. The NPA results were then compared with an explicit measure of the NF-κB complex activity provided by its nuclear translocation.

Activation of the stress- and immune-response transcription factor NF-κB (nuclear factor kappa-light-chain enhancer of activated B cells) has been well-defined as a major mediator of TNFα-induced signaling in a variety of systems [18,19]. NHBE cells were treated with four different doses of TNFα (0.1, 1, 10 and 100 ng/mL) and total RNA was collected for microarray measurement at four different times after treatment (30 minutes, 2 hours, 4 hours and 24 hours) (see Methods). All treatments were compared to time-matched mock-treated controls to obtain 16 contrasts (4 doses × 4 time points).

**Figure 2:**
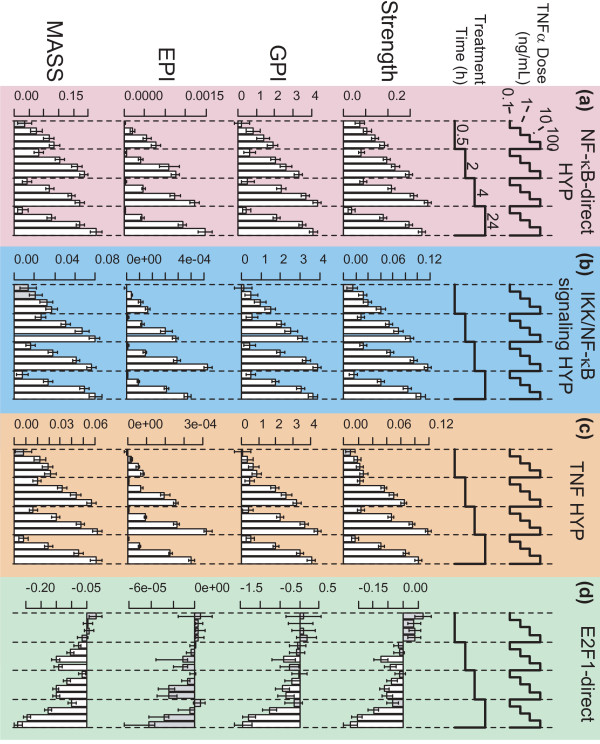
**HYP scores for TNFα-treated NHBE cells.** Transcriptomic data from TNFα-treated NHBE cells was scored using each scoring method (Strength, GPI, EPI, and MASS) for **(a)** the NF-κB-direct HYP, **(b)** the IKK/NF-κB signaling HYP, **(c)** the TNF HYP, and **(d)** the E2F1-direct HYP. Error bars represent the 95 % confidence interval as determined by the Uncertainty statistic, and scores that failed the Specificity criterion (Specificity *p*-value > 0.05; 1000 comparable HYPs) are shaded in gray.

**Figure 3:**
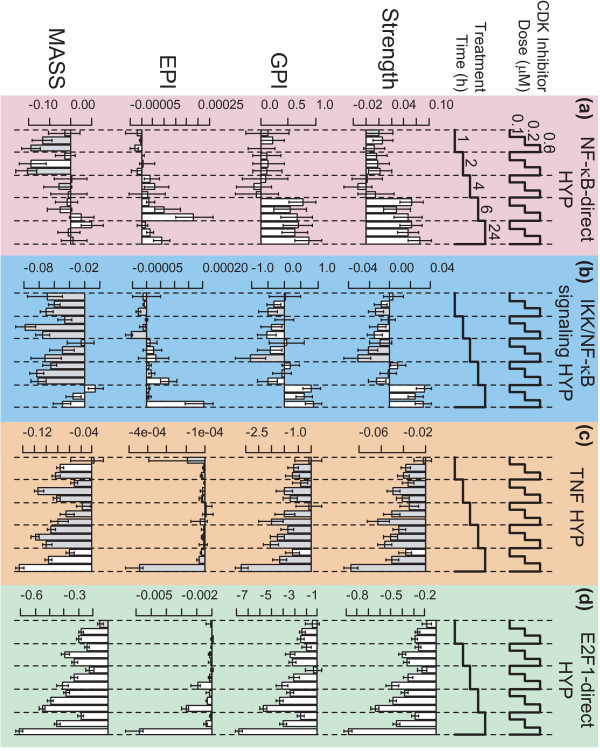
**HYP scores for CDK inhibitor-treated HCT116 cells.** Transcriptomic data from HCT116 colon cancer cells treated with the CDK inhibitor R547 was scored using each scoring method (Strength, GPI, EPI, and MASS) for **(a)** the NF-κB-direct HYP, **(b)** the IKK/NF-κB signaling HYP, **(c)** the TNF HYP, and **(d)** the E2F1-direct HYP. Error bars represent the 95 % confidence interval as determined by the Uncertainty statistic. Scores that failed the Specificity criterion (Specificity *p*-value > 0.05; 1000 comparable HYPs) are shaded in gray.

Each amplitude scoring method was investigated using a HYP created to be a specific measure of NF-κB activation, the NF-κB-direct HYP (Additional file 1). This HYP is composed of 155 genes (curated from 247 distinct references, some genes being supported by more than one reference) known to be directly regulated by NF-κB (genes whose expression is controlled in an NF-κB-dependent manner and whose promoter sequences are directly bound by NF-κB). Each scoring method showed the same pattern of response to TNFα, having demonstrated a dose-dependent response at all times, and a time-dependent response that generally saturated at later times (2 hours or 4 hours, depending on the scoring method; Figure 2a). However, there were some differences between the score patterns for each scoring method. The closely-related Strength and GPI methods produced almost indistinguishable patterns of response, suggesting the contributions from noise were balanced in this experiment (see Table 1). The EPI method was qualitatively different from Strength and GPI in that EPI scores continued to increase from 2 hours to 4 hours to 24 hours, while Strength and GPI scores plateaued from 4 hours to 24 hours. Also, the EPI method produced near-zero scores for 0.1 ng/mL TNFα. In general, EPI scores for other HYPs and data sets also appeared to reduce to 0 (or near to 0) scores that trended relatively lower by other methods (Figures 2 and 3). The MASS method qualitatively differed from Strength and GPI primarily at the 4 and 24 hour time points, with Strength and GPI scores increasing from 2 hours to 4 hours and plateauing from 4 hours to 24 hours, while MASS scores plateaued from 2 hours to 4 hours and increased from 4 hours to 24 hours. Strength and GPI scores met the Specificity criterion (Specificity *p*-value < 0.05) for all conditions. The lowest dose and earliest time point for MASS, and the lowest dose for all but the 2 hour time point for the EPI method, were found to not be specific to the NF-κB-direct HYP.

**Figure 4:**
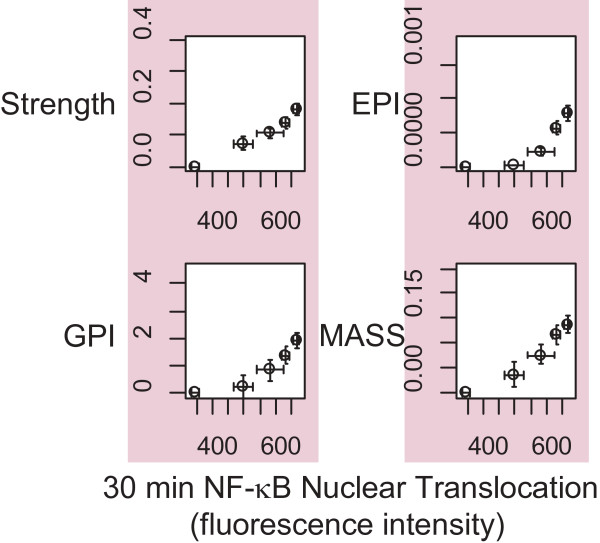
**HYP scores versus NF-κB nuclear translocation.** The NF-κB-direct HYP scores at 30 minutes for each amplitude scoring method (Strength, GPI, EPI and MASS) plotted against NF-κB nuclear translocation at 30 minutes. Score error bars represent the 95 % confidence interval as determined by the Uncertainty statistic. Error bars in NF-κB nuclear translocation represent the standard deviation of the mean nuclear translocation for three different fields of view of the same population of cells. The Pearson correlation between nuclear translocation and NPA score was 0.98 for Strength, 0.92 for GPI, 0.85 for EPI, and 0.97 for MASS.

Next, NF-κB-direct HYP scores were compared to NF-κB nuclear translocation. Upon activation, NF-κB is transported into the nucleus where it acts to regulate the expression of many genes [18,19]. A series of feedback loops then lead to the subsequent translocation of NF-κB back to the cytoplasm, and this oscillatory cycle continues several times [19]. Because NF-κB oscillations occur with slightly different periods in different cells in the population, the first oscillation is the most reliable population-measure of NF-κB activation. Although the time of the first oscillation depends on dose, 30 minutes after TNFα treatment is a realistic time to measure NF-κB nuclear translocation for the doses used here (Additional file 2) [19]. Each scoring method produced a monotonic, and in some cases nearly linear, relationship between score and nuclear translocation, with Pearson correlation coefficients between 0.85 and 0.98 for the different scoring methods (Figure 4). Interestingly, this dose-dependent relationship was preserved at different times after TNFα treatment (Additional file 3). These findings demonstrate that the HYP-based NPA scores can quantify NF-κB transcriptional activity.

### NPA scoring of additional HYPs can quantitatively assess response to TNFα

The effects of HYP size and composition were investigated. First, the effect of hand-selecting a set of measurements that are known to be modulated by NF-κB specifically in a TNFα-dependent manner was assessed. A HYP was constructed from a set of 20 genes that were previously measured via RT-PCR to assess NF-κB activity in response to TNFα treatment in 3T3 mouse fibroblast cells (omitting 2 genes that have no direct human ortholog) [19]. These genes were measured as perturbed by TNFα in 3T3 cells upon dosing with TNFα (10 different concentrations spanning 100 ng/mL to 0.005 ng/mL) over a 12 hour time course. This HYP produced a very similar pattern of activation to the NF-κB-direct HYP (Additional file 4), suggesting that inclusion of genes whose TNFα-dependent expression has not been directly verified does not have a detrimental effect on the quality of the HYP score.

Next, the effects of using HYPs derived from upstream biological entities that are less proximal to the measurement were investigated. To do so, two additional HYPs were constructed: the IKK/NF-κB signaling HYP (Additional file 5), which is composed of 992 genes (curated from 414 different references) that are known to be modulated by perturbation of proteins in a causal network model of signaling from the IκB kinase (IKK) proteins to NF-κB activation (Additional file 6); and the TNF HYP (Additional file 7), which is composed of 1741 genes (curated from 589 different references) that are known to be modulated by treatment of cells with TNFα. The IKK/NF-κB signaling HYP was generated by first constructing a causal network model of IKK/NF-κB signaling, and then transforming it into a single HYP by the aggregation procedure illustrated in Figure 1b (see Methods). Whereas the NF-κB-direct HYP is composed entirely of genes whose expressions were directly controlled by a single transcription factor (NF-κB), each of these two HYPs contains genes whose direct transcriptional controller is not necessarily known. The expression of these genes may be controlled by transcription factors not involved in constructing the HYP. For example, genes in the IKK/NF-κB signaling HYP are known to be modulated by perturbation of proteins in the IKK/NF-κB signaling causal network model, but some of these genes could be regulated as secondary effects caused by altered expression of a smaller subset of genes that are directly modulated by NF-κB. Also, TNFα is a ligand and therefore does not directly mediate transcription of any genes. Treatment of cells with TNFα results in activation of a myriad of transcription factors, any of which may directly or indirectly (for example, through autocrine signaling) alter the expression of each gene in the TNF HYP.

The IKK/NF-κB signaling HYP and TNF HYP give insight into the behaviors of HYPs at different levels of proximity to the measurements. The IKK/NF-κB signaling HYP is primarily composed of genes that are regulated (either directly or indirectly) by NF-κB (Additional file 6), and it produced a pattern of response that is very similar to the NF-κB-direct HYP (Figure 2b). This similar pattern of response suggests that there is not a large difference between the population-level behavior of genes that are known to be *directly* regulated by a transcription factor and the behavior of genes where knowledge of *direct* regulation is unknown. The time- and dose-dependent response that was seen for the NF-κB-direct HYP appears somewhat less robust in the TNF HYP (Figure 2c), for example at the 30 minute time point, but again the four methods produced very similar responses. Thus, although the general pattern of response was well-preserved among the HYPs, minor but noticeable differences in response can be observed in HYPs that are less proximal to the measurements.

### NPA scoring detects limited cross-talk between NF-κB and cell cycle signaling

To assess the ability of HYPs to respond specifically to relevant TNFα signaling perturbations, a HYP was constructed for a key cell-cycle component, the transcription factor E2F1 (Additional file 8), with the assumption that E2F1 is a less direct effector of TNFα signaling compared to NF-κB. The E2F1-direct HYP is composed of 80 genes (curated from 54 different references) known to be directly regulated by E2F1 (expression controlled by E2F1 and promoter sequence bound by E2F1). The E2F1-direct HYP showed a dose-dependent decrease in score for MASS at the 2, 4, and 24 hour time points, and for Strength and GPI at the 24 hour time point (Figure 2d). Consistent with this predicted decrease in cell cycle progression, CellTiter-Glo® measures of cell number found no appreciable increase in cell number after 24 hours of TNFα treatment (Additional file 9). Further verification of these conclusions could be performed, for example, by measuring the cell cycle progression of the sample populations via flow cytometry.

In order to provide a comparison of NPA results for biology not directly related to NF-κB signaling, the NPA response of the four HYPs introduced above (NF-κB-direct, IKK/NF-κB signaling, TNF, and E2F1-direct) were assessed in response to inhibition of cell cycle progression via a CDK inhibitor. Specifically, a publicly available microarray data set was used for treatment of HCT116 colon cancer cells with three different concentrations of the CDK inhibitor R547 (GSE15395) [15] (Figure 3). All four NPA methods demonstrated dose- and time-dependent decreases in the E2F1-direct HYP score at the 4 hour, 6 hour, and 24 hour time points. The TNF HYP showed a similar pattern of response as the E2F1-direct HYP, however few of the scores passed the Specificity criterion. This suggests that some of the genes in this HYP are cell cycle regulated, but are not sufficient in number to pass the Specificity criterion. In contrast, the NF-κB-direct and IKK/NF-κB signaling HYP scores did not display this same dose- and time-dependent pattern, indicating that these focused HYPs potentially contain few cell cycle regulated genes. However, the scores for the two NF-κB HYPs showed significant increases at the 6 hour time point (NF-κB-direct HYP) and the 24 hour time point (NF-κB-direct and IKK/NF-κB signaling HYPs), suggesting that NF-κB may perhaps be activated by cell cycle arrest (for example, [20]). Furthermore, the pattern of scores for the NF-κB-direct and IKK/NF-κB signaling HYPs were significantly different for the CDK-inhibition data set, indicating that these HYPs will not produce near-identical patterns of scores under circumstances where NF-κB is not activated or is perhaps regulated in a complex manner.

## Discussion

Previous work has demonstrated the utility of exploring the reverse causal interpretation of large scale data sets using HYPs as opposed to reasoning downstream from the data [12-14]. The ability to deduce the degree of activity for a broad spectrum of biological processes, afforded by an extensive causal knowledgebase, would provide enormous potential for facilitating biological characterization and yield an even deeper mining of information from large-scale data. This approach offers the potential to quantify responses of biological systems to anything from toxicity and disease processes to therapeutic benefit. This study successfully demonstrated the use of the causal connections provided by an appropriate knowledgebase as the basis for quantifying the activity degree of specific biology from high-throughput data. This quantitative application of HYPs, representing possibly complex network models, to experimental data measuring treatment-induced perturbations is called Network Perturbation Amplitude (NPA) scoring.

### Causal directionality is key for the HYP framework

For all NPA metrics, the proper scoring of a HYP is dependent upon the directionality (signs) of the causal influences linking the upstream biological entity represented by the HYP to the downstream genes whose expression it regulates. The knowledgebase harbors information about the specific signs (positive or negative) of the regulation exerted by the entity represented by the HYP on the expression of each of the downstream genes. The logic for incorporating differential gene expression measurements into an NPA score based on a knowledgebase-specified directional blueprint can be made via arguments against two specific alternative scoring schemes. The case of scoring an activity without taking into account the sign of causal influence in the HYP can make sense if the HYP represents a transcription factor that always activates or represses genes. However, if there are downstream genes in a HYP that are controlled in an opposite manner to the others, the error of an activity score based on an assumption of a single sign becomes apparent: the score contribution of genes that are known to be negatively regulated within a HYP might cancel, instead of add to, the score contribution of genes that are known to be positively regulated within a HYP. An alternative tactic would be to incorporate the absolute values of the differential expression for each gene. This has the problem of always producing positive scores for a HYP as well as artificially inflating scores: genes that change in a manner opposite of how a HYP is known to control a gene would add to a HYP activity score rather than detract from it. Standard gene enrichment analyses usually ignore regulation signs when scoring pathways [21,22], but like some newer gene enrichment methods [23], NPA assessment methods integrate both types of causal signed relationships of the biology to the measurements for their output.

### HYPs can be rapidly constructed from an appropriate knowledgebase

HYPs were constructed from the Selventa Knowledgebase, a database of causal biological knowledge that allows rapid creation of HYPs for any biological process, entity, or causally consistent network model that is adequately connected to gene expression changes (see Methods). The TNF and E2F1-direct HYPs were created from this knowledgebase without any additional literature or experimental investigation. For the NF-κB-direct HYP, because the content of the knowledgebase was biologically too limited in this context, additional genes that are directly regulated by NF-κB were mined from the literature and added to the knowledgebase as causal relationships. The additional knowledge concerning the direct effects of NF-κB was necessary to ensure a broadest representation of NF-κB biology. To construct the IKK/NF-κB signaling HYP, a network model was built by assembling causal relationships between relevant entities that were represented in the Selventa Knowledgebase. Similarly, HYPs and network models can integrate information from other sources besides the Selventa Knowledgebase, including literature articles and curated databases, as long as this information can be interpreted as signed causal relationships. The boundaries of HYPs and network models are defined during their construction (see Methods). For this study the model boundaries were chosen based on the biology known to be associated with the TNFα treatment of NHBE cells. In a case where the expected biology is unknown, a process of identifying biology is required to determine the most appropriate network models and HYPs to score. Such an exploratory perspective can be provided by evaluating the resulting HYPs using the RCR approach [1], which provides a statistical assessment of whether the activation of a biological entity is consistent with measured data, as previously described [12-14].

Building the IKK/NF-κB signaling network model afforded the ability to aggregate the gene expression measurements that underlie all the individual HYPS of a specific NF-κB network and provide a single score for that network. However, in condensing a complex model into a single score, there are caveats to consider. Gene expressions that have an ambiguous relationship to the network (both causally positively and negatively regulated) must effectively be removed for scoring purposes. These ambiguities affect approximately 6 % of the downstream genes of the aggregated IKK/NF-κB signaling HYP, which is similar to the case of single HYPs: the NF-κB-direct and the TNF HYPs contains approximately 4 % and 7 % ambiguous downstream genes, respectively. Their actual impact on the NPA results is expected to remain limited (see below).

Additionally, resolution with regard to which individual entities of the network model are being perturbed is also diluted in the overall network score. Thus, when generating an aggregated HYP for a network model, key information about the network is not explicitly available, and it is important to keep these features in mind when interpreting NPA scores (see below for a further discussion of the methodological perspectives). However, despite these caveats, the IKK/NF-κB signaling HYP produced a near-identical pattern of response as the NF-κB-direct HYP, and thus is also correlated with the measured physiological endpoint, NF-κB activation.

This finding that similar NPA results were obtained using HYPs featuring different characteristics highlights an essential aspect of this work: using the same network model for calculating the NPA scores of the various experimental conditions to be compared (e.g., for all TNFα doses and post-treatment times) provides results that are robust against having exhaustively captured the perturbed biology in the network model used for NPA scoring. This aspect is fundamental, especially given the practical impossibility of constructing networks models capturing all of the biological processes potentially perturbed in a given experiment. It is also exploited when constructing network models describing processes that are sufficiently generic, e.g., cell proliferation or cellular stress [16,17], so that they can be meaningfully applied in a variety of experimental situations.

The robustness of the results also preserves the validity of the NPA approach against the possibility of HYPs and network models evolving slightly due to the constantly improving understanding of the biological processes they describe. This property was concretely tested with a simple step-back calculation consisting of randomly removing edges to the HYPs and comparing the corresponding NPA results to the original ones. The results demonstrated a remarkable robustness: typically, after removal of 20 % of their downstream genes, the four HYPs used in this work returned GPI profiles that correlated extremely well with their original values shown on Figure 2 (Spearman correlations of 0.99 ± 0.01 obtained on 1000 samples). Therefore reasonable future additions to the Selventa Knowledgebase are not expected to significantly impact the NPA results presented in the work. As a corollary, these robustness considerations also support the choice of discarding the downstream genes expressions that have an ambiguous relationship to the HYP upstream entity. Examination of Additional file 1, Additional file 5, Additional file 7, Additional file 8 showed that the fraction of ambiguous downstream genes never exceeds 10 %, which clearly indicates that this effect does not affect the NPA results.

### NPA scoring methods accurately assess biological activation

Four different algorithms were developed to quantify the amplitude of perturbation of a HYP. Each method employs a different approach to evaluate the degree of perturbation between two experimental measures for a given HYP (see Table 1 and Methods). Despite their differences, the four methods generally produced similar qualitative results, suggesting that each NPA scoring method is able to effectively quantify the changes in activity of the underlying biological processes. This claim is supported by the fact that the NPA scores correlated well with NF-κB nuclear translocation, a standard measure of NF-κB activity (correlation coefficients between 0.85 and 0.98). This correlation further validates our method of constructing HYPs from a database of prior knowledge.

Future work will confirm the circumstances in which each method is expected to be the most appropriate. For example, the MASS algorithm was developed to use absolute measurement technologies such as an absolute transcript count offered by quantitative next generation sequencing. Given more appropriate measurement technology, the MASS method may be more applicable in circumstances where small differential expressions in a set of highly expressed genes have a dominant effect over large differential expressions in a set of weakly expressed genes. On the other hand, the GPI algorithm, and to an even larger extent the EPI algorithm, down-weight the contributions from genes with poor statistical significance, favoring small sets of strongly differentially expressed genes rather than large sets of weakly differentially expressed ones. From this point of view, the Strength algorithm is unbiased since it contains no weighting factors. However, because an NPA score represents a condensed view of the biology underlying a HYP, the ability to assess the amplitude of its perturbation with complementary NPA methods also highlights which conclusions are robust versus which conclusions may be specific to a particular NPA method. For example, the four NPA methods supported the same time- and dose- dependent NF-κB activation in response to TNFα (Figure 2a), whereas only Strength and GPI suggested NF-κB activation in response to CDK inhibition (Figure 3a).

There are some important considerations when using NPA scoring methods to evaluate HYPs. Scores are meant to be directly compared between different treatment versus control contrasts when using the identical HYP. Scores cannot be quantitatively compared between two different HYPs without first verifying that the relationship between a change in the activity of the HYP’s upstream entity and the resulting change in the expression of downstream genes is conserved between the two HYPs. In general, this relationship is not expected to be conserved due to differences in the dynamic range of expression of individual genes that compose each HYP. Additionally, a HYP with a higher number of downstream gene expressions may be expected to represent broader biology than a smaller HYP, and thus in any given experiment, a smaller fraction of genes in the larger HYP may be perturbed, resulting in a lower score than a smaller HYP. However, additional statistical power is gained in the Uncertainty and Specificity statistics with increasing number of downstream gene expressions in a HYP, such that the weaker scores of larger HYPs can be just as significant and meaningful as higher scores from smaller HYPs.

Although scores cannot be directly compared between two different HYPs, the pattern of scores across a set of contrasts can be qualitatively compared. Likewise, the absolute magnitude of the NPA scores should not be directly compared between two amplitude scoring methods, but the pattern of scores across NPA scoring methods can be qualitatively compared, keeping in mind that the scoring methods may be assessing different aspects of the contrasts.

The NPA score represents an abstracted measure of the data represented in the HYP. The score captures the amplitude of the perturbation of a HYP, but does not capture which genes in the HYP most strongly contribute to the score. For example, of the 20 genes that contribute most to the IKK/NF-κB signaling HYP score upon TNFα treatment (100 ng/mL, 24 hour), only one is in common with the 20 genes that contribute most to the IKK/NF-κB signaling HYP upon CDK inhibition (0.6 μM, 24 hour). Given that the NF-κB-direct HYP consists of only 155 genes, this suggests that there is a significant difference in the biology represented by the NF-κB-direct HYP scores in these two cases.

### Uncertainty and specificity of NPA scores

Uncertainty estimates the confidence interval of each NPA statistic, and therefore also tests the nullity of the score accounting for the experimental error. The Specificity statistic gives a measure of whether the score is dependent on the expression of specific genes in the HYP, or is instead dependent on a particular property (the likelihood of modulation) of the ensemble of gene expressions in the HYP. Although this definition of Specificity is useful, there are some important points to ensure that Specificity is interpreted appropriately. First, a weak Specificity does not mean that the score fails to accurately characterize the amplitude of the process described by the HYP. Rather, it means that many other *comparable* HYPs would obtain a similar score. For example, a very weak score (approximately zero) for a transcriptomic HYP is likely to have a weak Specificity because the majority of the genes on a microarray are unchanged under most conditions. Thus, any random assortment of genes in a HYP might produce a similarly low score. Weak Specificity for low scores could therefore be an indication that the genes in the HYP are not sufficiently perturbed. Alternatively, a high score with a weak Specificity does not indicate that the process measured by the HYP is not perturbed. Rather, it indicates that comparable gene expressions are perturbed to a similar extent, suggesting that other processes with comparable HYPs are likewise perturbed, and thus the score cannot be attributed with high probability to the process represented in the HYP. For example, the fact that the pattern of Strength scores for the TNF HYP in the CDK inhibitor experiment is similar to the pattern of Strength scores for the E2F1-direct HYP suggests that the TNF HYP may contain some genes that are cell cycle controlled (Figure 3). However, this number of genes is not sufficient to distinguish this score from the “background” of scores for comparable HYPs, as only one of the fifteen TNF HYP Strength scores met the Specificity criterion. In fact, 32 genes are common to the TNF HYP and the E2F1-direct HYP, which constitutes more than a third of the E2F1-direct HYP, but only one fortieth of the TNF HYP. Methods such as Network Component Analysis [24,25] could possibly be adapted to resolve overlaps between HYPs by assigning shared gene expressions to the most statistically likely HYP, potentially increasing the precision of each HYP and modulating score Specificity appropriately.

Together, the Uncertainty and Specificity statistics enable the identification of non-specific and non-significant scores in HYPs when scored against unrelated perturbations. These statistics demonstrate that TNFα treatment of NHBE cells only has a significant effect on cell cycle progression when the dose is above 0.1 ng/mL, and that this effect takes two-to-four hours to appear. Also, these statistics support the conclusion that some NF-κB-regulated genes are upregulated at 6 and 24 hours after CDK-inhibition in HCT116 cells, but likely not at 4 hours or earlier.

### Potential applications beyond the comparative assessment of biological impact

The NPA approach developed in this work aims at quantifying the treatment-induced perturbations of the biological processes described by causal network models. It enables the comparative assessment of the biological impact from high-throughput data in response to given stimuli. However the NPA approach could be also used in more exploratory perspectives. For instance, by applying NPA scoring to each HYP in a causal network model, rather than constructing and scoring a single aggregated HYP for the model (Figure 1b), differences in activation across a model could be investigated. Scoring individual HYPs within a model instead of the larger aggregated model HYP presents a tradeoff of increased granularity of information at the expense of statistical robustness, due to the smaller sizes of the HYPs being scored. Another possibility would be to use the NPA scores and their companion statistics to identify which processes are potentially activated in response to a given perturbation, and thus help guide the construction of a HYP or of a causal network model that capture the relevant perturbed biology.

Finally, NPA scores could be used as a supplementary source of information in studying different types of mathematical models of regulatory networks. The fact that NPA scores provide quantitative measurements for the response of entities that are not explicitly measured or measureable can be exploited in the construction, calibration, or evaluation phases of such models. For instance, in the case of TNFα-treated NHBE cells considered in this study, the NPA scores provide direct quantitative measurements of the inflammatory response of the system, a quantity that would be difficult to access in the absence of the NF-κB nuclear translocation measurements performed in this work.

## Conclusions

NPA is an integrated approach that combines high-throughput experimental data and a knowledge-driven HYP, which provides measurable quantities causally affected by a targeted biological process, to quantify the activity changes of that process relative to a control (non-perturbed) state of the system. The utility of the NPA method lies in the synergy of on-demand HYP generation from an extensive causal knowledgebase with a continuous measure of its activity change. Four NPA scoring methods with complementary strengths performed similarly in this study, but individually have the potential to wield distinct advantages for specific circumstances. Additionally, qualifying NPA statistics enabled effective use of these scoring metrics and can be applied to similar methods developed elsewhere. When applied to TNFα- and CDK inhibitor-treated cell microarray data, NPA scores for NF-κB and cell cycle networks correlated with expected dose response relationships and specific measured pathway outputs. NPA scoring also suggested possible cross-talk between NF-κB activation and the cell cycle that could be investigated experimentally. With a broad spectrum of biology available to score within the Selventa Knowledgebase, NPA metrics and statistics can be used to assess amplitude of perturbation on many orders, from a single molecule to that of a complex, higher-order causal network model representing complex biological processes. This approach enables a quantitative, systems-wide understanding of the biological mechanisms leading to diseases. This is the first step towards the development of computational tools designed to comparatively measure any perturbation, including exposure to toxic substances, the effects of drug treatment, or patient stratification by individual biology.

## Methods

### Experimental procedure

Normal human bronchial epithelial cells (Lonza Walkersville, Inc.) were cultured in standard growth medium (Clonetics medium, Lonza Walkersville, Inc.). Cells were either treated with TNFα (Sigma) or a vehicle control (HBSS), and then harvested after the desired treatment length (30 minutes, 2 hours, 4 hours, or 24 hours). Cells were immediately put on ice and split into three technical replicates from which total RNA was extracted using RNeasy™ Microkit (Qiagen). The processed RNA samples are then hybridized to Affymetrix U133 Plus 2.0 microarrays. Cell viability and cell counts were controlled for all conditions after 24 hours with CellTiter-Glo® assay (Promega). NF-κB nuclear translocation was measured using Cellomics NF-κB Activation HCS Reagent Kit (Thermo Scientific).

### Data processing and algorithm implementation

Data processing and NPA methods were implemented in the R statistical environment [26]. Raw RNA expression data was analyzed using the *affy* and *limma* packages of the Bioconductor suite of microarray analysis tools available in the R statistical environment [27,28]. Robust Microarray Analysis (RMA) background correction and quantile normalization were used to generate probe set expression values [29]^a^. An overall linear model was fit to the data for all groups of replicates, and specific contrasts of interest (comparisons of “treated” and “control” conditions) were evaluated to generate raw *p*-values for each probe set on the expression array. Raw *p*-values were subsequently corrected for multiple testing effects using Benjamini-Hochberg false discovery rate (FDR), as described hereinafter.

Probe sets were matched to RNA Abundance nodes in the Selventa Knowledgebase (see below) using the *HG-U133_Plus_2.na30* probe set mappings and the following criteria. First, only “at” or “s_at” probe sets were considered. Second, probe sets that mapped to multiple genes were discarded. Third, when multiple probe sets mapped to the same gene, preference was given to “at” probe sets over “s_at” probe sets. Finally, when there still remained multiple probe sets mapped to the same gene, the probe set with the lowest geometric mean FDR-corrected *p*-value across all contrasts of interest was selected. A linear model was then re-fit for all groups of replicates to only those probe sets that mapped to RNA Abundance nodes in the knowledgebase, and FDR-corrected *p*-values were recomputed as described in this section.

### The Selventa Knowledgebase

The Selventa Knowledgebase is a comprehensive repository containing over 1.5 million nodes (biological concepts and entities) and over 7.5 million edges (assertions about causal and non-causal relationships between nodes). The assertions in the knowledgebase are derived from peer-reviewed scientific literature as well as other public and proprietary databases (Figure 1a). Specifically, each assertion describes an individual experimental observation from an experiment performed in a human, mouse, and rat species context, either in vitro or in vivo. Assertions also capture information about the referring source (e.g. the PubMed ID (PMID) for journal articles listed in MEDLINE), as well as key contextual information including the species (human, mouse, or rat) and the tissue or cell line from which the experimental observation was derived. An example causal assertion is the increased transcriptional activity of NF-κB (nuclear factor kappa-light-chain-enhancer of activated B cells) causes an increase in the mRNA expression of CXCL1 (Chemokine (C-X-C motif) ligand 1) [HeLa cell line; Human; PMID 16414985]. The knowledgebase contains causal relationships derived from healthy tissues and disease areas such as inflammation, metabolic diseases, cardiovascular injury, liver injury, and cancer.

### Constructing HYPs and causal networks models

In addition to edges that enable the construction of HYPs (i.e., causal relationships between one upstream entity and its downstream measurables; Figure 1a), the Selventa Knowledgebase also contains literature-curated relationships between the upstream entities of different HYPs. These edges can be assembled to construct larger causal network models describing more exhaustively the biological processes under consideration (Figure 1b).

The principles guiding the construction of the HYPs used in this work were pragmatic (e.g., the NF-κB-direct, TNF, and E2F1-direct HYPs, see Results). The downstream measurable nodes derived from the Selventa Knowledgebase in an automated manner are gene expressions (“exp(…)” nodes on Figure 1a) that have been shown to be causally linked to the HYP upstream entity in a variety of experiments, cell types, and even species. Ideally, this context information stored in the knowledgebase could be leveraged to construct HYPs using only knowledge derived from the cell type and perturbation under current study. However, despite the large amount of information in the Selventa Knowledgebase, there was generally insufficient material from similar contexts to enable automated construction of context-specific HYPs. Therefore context-based HYP filters were not used in this work. This choice is supported by the fact that HYPs with a larger number of downstream genes increase the likelihood that a number of the downstream genes in the HYP will be relevant to the system being investigated. Furthermore, the positive and negative score contributions from irrelevant downstream genes are expected to balance each other and produce a background noise averaging to zero and thus not to affect the final NPA results. This assumption was confirmed *a posteriori* by the comparisons of the Strength and GPI NPA scores (see Results).

Causal network models (e.g. the IKK/NF-κB signaling network model, see Results) are constructed by manually assembling causal relationships connecting HYP upstream entities derived from the Selventa Knowledgebase (Figure 1b). While context information is not taken into account during the process of HYP construction, the process of network model construction is guided by the cell type and perturbation relevance of each causal connection in order to get a high-quality outcome. In this study the boundaries of the IKK/NF-κB signaling network model were fixed so that signaling in NHBE cells in response to TNFα treatment was accurately described. A more detailed discussion on the construction of causal network models is given under the construction process of the “Literature Model” in two published studies involving similar network types [16,17].

It is important to note the difference between the knowledge-based causal network models used in this work and the networks constructed from expression data using network inference approaches [30]. Although both are “causal”, their content is fundamentally different. Knowledge-based network models are constituted of literature-curated causal relationships between biological entities, for which the NPA approach enables “backward” deduction of the activity changes (or perturbation) from differential gene expression data. The expression data-based inferred networks describe gene-gene interactions that are “forward” deduced from the expression values of the corresponding genes and measured over a large collection of experimental conditions. Therefore, whereas linked genes are interacting in the networks inferred from expression data, gene expression nodes (i.e. “exp(…)” nodes in Figure 1) that are connected via a HYP in a knowledge-based network model are under the influence of a common upstream entity (e.g. the transcriptional activity of NF-κB for the NF-κB-direct HYP).

### Constructing a HYP from a causal network model

A causal network model is composed of multiple causally-linked nodes that are biologically related, including HYPs [16,17]. In order to generate the corresponding aggregated HYP, a reference node must first be selected within the network model. The reference node can be any entity in the network whose level or activity is positively related to the activity of the network as a whole (as opposed to, for example, an inhibitor whose activity may be negatively related to the network activity). Next, the causal relationship between each node in the model and the reference node is determined. This can only be done by first requiring that the model be “causally consistent” (see below). The signs of regulation of downstream measurable entities (here, gene expressions) for each node in the model are adjusted based on the relationship between that model node and the reference node. For example, the signs of the downstream gene expressions for a model node that has a positive causal relationship with the reference node (i.e., that node is expected to be positively regulated when the reference node increases) are maintained. On the other hand, the signs of the downstream gene expressions for a model node with a negative causal relationship with the reference node (i.e., that node is expected to be negatively regulated when the reference node increases) are inverted. All the downstream gene expressions and their signs are then assembled into a single HYP (Figure 1b), and downstream gene expressions with contradictory signs (from multiple model nodes) are omitted from the aggregated HYP.

For a network model to be causally consistent, for an increase in any node in the model, it must be possible to unambiguously map a sign of “positive regulation” or “negative regulation” on every other node in the model by following the causal relationships that connect the nodes. For example, any model with negative feedback loops cannot be used to construct an aggregated HYP. Biological interpretation can be used to resolve ambiguities to construct causally consistent models by considering what process is being scored by the HYP, and in what sign each node is effectively related to the reference node. For example, the node where a negative feedback connects back to the model has a particular relationship with the process being scored, and although the negative feedback may regulate this node, it should not change this relationship. Thus, the connection between the negative feedback loop and this node can be removed from the model to obtain causal consistency in a manner that is congruent with our biological expectations. Such a biology-driven procedure to build causally consistent network models implies a careful definition of the network model boundaries. Resolving the potential sign ambiguities in its nodes involves additional refinements in delimiting the biological processes described by the model.

### NPA scoring algorithms

#### Strength

The Strength NPA scoring method was developed as the simplest measure of HYP amplitude – the weighted mean of the measurement differences (here, gene log_2_ differential expressions) where the weighting factors are the signs of the measurable downstream entities in the HYP:

(1) $$Strength=\frac{1}{N}\sum_{i=1}^{N}s_{i\;}\cdot \beta_{i}$$

where *β*_*i*_ is the log_2_ differential expression (i.e., the log_2_ fold change) of the *i*^th^ gene in the HYP, and *s*_*i*_ is the sign (+1 for positive regulation and −1 for negative regulation) of the *i*^th^ gene in the HYP, and *N* is the number of measurable downstream gene expressions in the HYP. This translates to the sum of log_2_ differential gene expressions with positive regulation in the HYP minus the sum of the log_2_ differential gene expressions with negative regulation in the HYP, divided by the total number of gene expressions in the HYP. Thus, a positive Strength score indicates that the HYP’s downstream gene expressions and their signs of regulation are matched within the data, and the process described by the HYP is upregulated in the treated condition compared to the control condition. A zero Strength score indicates that the process is unchanged, and a negative Strength score indicates that the process is downregulated.

The Strength scoring method uses data for all measured downstream gene expressions in the HYP, regardless of data quality. However, the differential expression values used by the Strength method may be dominated by noise for low absolute measures of the control condition, and thus may provide unreliable data (which should be evidenced by high uncertainty). The Strength method assumes that this noise is evenly distributed and that there are a sufficient number of measurable downstreams in the HYP such that measurement noise is averaged out across all downstreams.

#### Geometric perturbation index

A HYP can be seen as a unit sign vector *ŝ = (1,1,-1,1,…,-1)/√N* in the *N*-dimensional downstream measurable space (where each dimension represents a downstream measurable, here gene expression, of the HYP). The observed effect of perturbation on the downstream gene expressions is also a vector in this space. So geometrically, the amplitude of the perturbation in the HYP can be quantified by projecting the differential log_2_ expression vector *β* onto the hypothesis unit vector *ŝ*. However, the downstream measurements of a HYP come from a generic model. To deal explicitly with the specificity of data supporting an NPA score, each downstream is assigned a belief of activation, which is set to be the local false non-discovery rate (*fndr*_*i*_ *= 1-fdr*_*i*_) [31]. The false discovery rates *fdr*_*i*_ are obtained from the raw *p*-values using the Benjamini-Hochberg multiple testing corrections (see above). It is equivalent to weight the dimensions of the downstream gene expression space according to the belief of each differential expression and therefore consider a weighted scalar product to define the geometry of the gene expression space: ‹***ŝ|β***›_*fndr*_ ***= ŝ***^*T*^∙*diag*(***fndr***)∙***β***. Hence, the Geometric Perturbation Index (GPI) scoring method is defined as:

(2) $$GPI=\frac{1}{\sqrt{N}}\;\sum_{i=1}^{N}s_{i}\cdot fndr_{i}\cdot \beta_{i}$$

Note that Strength and GPI are closely related – GPI is normalized by *√N* rather than *N*. By weighting the differential log_2_ expression with false non-discovery rate, individual differential expression values for which there is little confidence are moved closer to zero (no change), while values for which there is stronger confidence are minimally decreased. A positive GPI score indicates an upregulation of the process described by the HYP, a zero GPI score indicates that the process is unchanged along the direction *s* of the HYP, and a negative GPI score indicates that the process is downregulated.

#### Measured abundance signal score

Both Strength and GPI quantify log_2_ differential values (i.e., log_2_ fold changes) of measurements in the HYP. However, there may be cases where an absolute change in mRNA, protein, or some other measurable physical quantity is a better measure of the biological effect on the process represented by a HYP. For example, an increase from 1 to 10 copies of an mRNA transcript (an absolute increase of 9 transcripts) may be less significant than an increase from 10 to 100 copies of the same transcript (an absolute increase of 90 transcripts). An NPA scoring method was devised to quantify absolute changes in entities that represent physical quantities, Measured Abundance Signal Score (MASS):

(3) $$MASS=\frac{\sum_{i=1}^{N}s_{i}\cdot (treated_{i}-control_{i})}{\sum_{i=1}^{N}(treated_{i}+control_{i})/2}$$

where *treated*_*i*_ is the measurement (not in log_2_ scale) for the *i*^th^ downstream measurable (here, gene expression) in the treated sample, and *control*_*i*_ is the measurement (not in log_2_ scale) for the *i*^th^ downstream gene expression in the control sample. The numerator of MASS represents the change in the absolute downstream gene expression quantities in a direction supporting an increase in the process described by the HYP. The denominator of MASS represents the average of the total absolute quantity of the downstream gene expressions. Thus, MASS can be thought of as quantifying the absolute change in the downstream gene expressions (corrected for the predicted sign *s*_*i*_ of each downstream in the HYP) compared to the total quantity of the downstreams. Rather than use the total quantity of the downstreams in either the treated or control condition alone, the average of the treated and control conditions was used to ensure that the MASS scoring method is symmetric about the experimental contrast (i.e., MASS(treated versus control) = −MASS(control versus treated)).

The MASS method is applicable to any measurement technique that quantifies physical measurables in a manner such that measurements are proportional to absolute quantities across all measurable entities (i.e., the measurements for different entities can be compared directly). It should be noted that for microarrays, this implementation is a proof of concept as there is only a loose correlation between the signal for different probe sets and the absolute amount of the transcripts [29,32].

#### Expected perturbation index

The Expected Perturbation Index (EPI) can be thought of as a smoothed version of the GPI. The rationale is to consider a HYP as a random variable over a compact interval [−*M,M*] containing all possible differential expression values (in log_2_ scale, typically *M* = 15 as gene expression signal saturates at 15). The downstream entities (here, gene expressions) of the HYP are used as evidence to construct the distribution *p*_HYP_ of the HYP: for any *φ* in [−*M,M*], the density of the HYP at *φ* is proportional to the total evidence of “correct predictions” *s*_*i*_*∙β*_*i*_ above *φ*. More precisely:

(4) $$p_{\mathrm{HYP}}(\varphi )\propto \{\begin{array}{cc} \frac{1}{N}\sum_{i\;\text{such that}\;s_{i}\cdot \beta_{i}>\varphi }\frac{\left|\beta_{i}\right|}{M} & \text{if }\varphi >\varepsilon \\ \frac{1}{N}\sum_{i\;\text{such that}\;s_{i}\cdot \beta_{i}<\varphi }\frac{\left|\beta_{i}\right|}{M} & \;\text{if }\varphi <-\varepsilon \end{array}$$

The normalization of the measure *p*_HYP_*(φ)∙dφ* is done through continuous interpolation of *p*_HYP_ between [−*ε*,*ε*], for *ε* sufficiently small (typically on the order of machine precision). The Expected Perturbation Index is then defined as being the expectation of the HYP with respect to the distribution *p*_HYP_ defined above:

(5) $$EPI=\frac{1}{2M}\int_{-M}^{M}\varphi \cdot p_{\text{HYP}}(\varphi )\cdot d\varphi$$

The EPI expresses the fact that high correctly predicted differential expressions *s*_*i*_*∙β*_*i*_ provide stronger evidence for the HYP than low ones and will therefore receive higher weights. The implementation is done by using the method of rectangle on the differential expression values, leading to the following formula to compute the EPI:

(6) $$\begin{array}{lll} EPI & = & \sum_{i\;\text{such that}\;{\left(s\beta \right)}_{i}>0}{\left(s\beta \right)}_{i}\cdot (\frac{1}{MN}\;\sum_{j=1}^{n_{+}}{\left(s\beta \right)}_{j})\cdot ({\left(s\beta \right)}_{i\;}-{\left(s\beta \right)}_{i-1})\\ & + & \sum_{i\;\text{such that}\;{\left(s\beta \right)}_{i}<0}{\left(s\beta \right)}_{i}\cdot (\frac{1}{MN}\;\sum_{j=1}^{n_{-}}{\left(-s\beta \right)}_{j})\cdot ({\left(-s\beta \right)}_{i\;}-{\left(-s\beta \right)}_{i-1}) \end{array}$$

where *(sβ)*_*i*_ is *s*_*i*_*∙β*_*i*_, *n*_*+*_ and *n*_*−*_ are the number of differential log_2_ expressions positively and negatively predicted by the HYP, respectively, subscripts *i* refers to the ascendant ordering of the |*s*_*i*_*∙β*_*i*_*|*_*,*_, and *β*_*0*_ *= 0*.

Note that the EPI is, in absolute value, bounded by *M*, and that the false non-discovery rates are not used. Indeed, high differential expression value are taken into account more often than lower ones, and this additional weighting to high differential expression enables a more specific measure of activity.

### NPA scoring statistics

#### Uncertainty

Each NPA score is a random variable. As such, the statistical significance of the NPA scores can be assessed by estimating confidence intervals around the score, or, equivalently, by evaluating the null hypothesis of the score equaling zero at a given type-I error risk *α* (often, and herein, *α* = 0.05). In the case of Strength and GPI, this task can be completed analytically, while in the case of MASS and EPI, a bootstrapping approach is necessary.

The theoretical distribution of the differential log_2_ expression is deduced from the estimation and test procedure used. In the case of t-statistics or moderated t-statistics (e.g., produced from a linear model by the *limma* R package), the (theoretical) distribution of the *β*_*i*_’s is assumed to be normal with variance *sd*_*i*_^*2*^ (having *df*_*i*_ degrees of freedom).

In this context, Strength becomes a random variable consisting of a weighted sum of independent (approximately) normal distributions. As a consequence, the distribution of the Strength statistic is (approximately) a normal distribution itself, with variance *Sd*_*Strength*_^*2*^ *= (1/N*^*2*^*)* ⋅ *∑ sd*_*i*_^2^. Hence, a t-statistic *t = Strength/Sd*_*Strength*_ can be derived, whose degrees of freedom *Df* are estimated with the Welch–Satterthwaite equation [33,34]. Therefore a (1-*α*)-confidence interval (e.g., 95 % confidence interval) for the Strength is given by:

(7) $$Strength\pm t_{\mathrm{Df}}^{\alpha /2}\cdot Sd_{\mathrm{Strength}}$$

In the case of the GPI, the situation is almost identical to the Strength, except for the additional dependence on the weighting factors *fndr*_*i*_. In turn, *fndr*_*i*_ depends on the non-adjusted *p*-value, and therefore on the original t-statistics for the *β*_*i*_, *t*_*i*_ *= β*_*i*_/*sd*_*i*_. The key step toward a test statistic for the GPI is the estimation of the variance of GPI. To this end, the variance is estimated through a first-order Taylor expansion to obtain:

(8) $$Sd_{\mathrm{GPI}}^{2}≅\frac{1}{N}\sum_{i=1}^{N}{\left(fndr_{i}+\beta_{i}\frac{\partial fndr}{\partial \beta_{i}}\right)}^{2}\cdot sd_{i}^{2}=\frac{1}{N}\sum_{i=1}^{N}\gamma_{i}^{2}\cdot sd_{i}^{2}$$

where:

(9) $$\gamma_{i}\equiv fndr_{i}+2\cdot \frac{fdr_{i}}{p_{i}}\cdot \frac{\left|\beta_{i}\right|}{sd_{i}}\cdot t_{df_{i}}(\frac{\beta_{i}}{sd_{i}})$$

Using the central limit theorem, an approximate t-statistic is derived as *t = GPI/Sd*_*GPI*_, whose degrees of freedom *Df* are again estimated by the Welch-Satterthwaite equation.

In the partial derivatives of the GPI with respect to *β*_*i*_, the derivative of *fndr*_*i*_ = 1- *fdr*_*i*_ involves two terms: one involving the derivative of the Benjamini-Hochberg adjustment factor (*fdr*_*i*_*/p*_*i*_), multiplied by *p*_*i*_ (the *p*-value for the differential expression of the *i*^th^ gene), and the other one involving the derivative of *p*_*i*_. The former is assumed to be zero, because the adjustment factor (*fdr*_*i*_*/p*_*i*_) is important only when the *p*-values *p*_*i*_ are small. The latter is computed analytically. Hence the (1-*α*)-confidence interval for GPI is given by:

(10) $$GPI\pm t_{\mathrm{Df}}^{\alpha /2}\cdot Sd_{\mathrm{GPI}}$$

In the case of MASS and EPI, a parametric bootstrapping of the distributions of *β*_*i*_ was used, taking advantage of the normality of the estimators *β*_*i*_ deduced from the statistical approach used to compute the differential gene expressions (see above). As the assumption for the application of percentile bootstrapping seems to be violated for MASS and EPI, the confidence interval was estimated using the bias-corrected percentile method [35,36].

#### Specificity

It is important to consider whether the computed NPA score is specific to the HYP of interest, or is a general property of the entire data set. For example, a score that indicated a two-fold increase in a given process holds less meaning if all measurements in the entire data set also increased two-fold. Thus, the Specificity statistic is computed as a means to identify scores that can be attributed with high probability to the specific biological entity or process represented by the HYP. Specificity is computed by assessing the likelihood of the following null hypothesis: “The amplitude score is not representative of the specific HYP, but instead is representative of a general trend in the data set that can be measured by any HYP that is comparable to the HYP of interest.” The first step to computing Specificity is to construct a set of comparable HYPs (see below). Next, an amplitude score is computed for each of these HYPs using the same data set. Finally, Specificity is computed as a two-tailed *p*-value by placing the amplitude score for the HYP of interest on the distribution of scores for the comparable HYPs (Additional file 10). Scores that have Specificity *p*-values less than 0.05 are considered to be scores that can be attributed with high confidence to the HYP of interest.

The key to computing the Specificity statistic is constructing relevant comparable HYPs. A simple comparable HYP could be composed of downstream measurable entities selected at random from the set of all measurable entities to produce a HYP of the same size as the HYP of interest. However, for some data sets including gene expression microarrays, many of the measurable entities are highly unlikely to change under any given circumstance (for example, genes whose expression are measured with ineffective probe sets). The HYP of interest will likely contain fewer of these entities than any “comparable” HYP because these entities are less likely to be affected by the process of interest, and are thus unlikely to be included in the HYP of interest. Therefore, a comparable HYP constructed by random sampling from all measurable entities will be biased towards showing a weaker (or no) change. This makes the amplitude score for the HYP of interest appear more unlikely to have occurred by chance given the null hypothesis – and thus be more specific – than might have otherwise been expected (Additional file 10).

The large body of causal knowledge available was used to construct more relevant comparable HYPs for transcriptomic data by first identifying the number of upstream controllers (distinct entities upstream of a gene in causal statements in the knowledgebase) for each gene in the entire data set, including the genes in the HYP. The number of upstream controllers reflects the number of different experiments or perturbations that caused the gene to be modulated, and acts as a naïve estimate for the likelihood of each gene being modulated in the current experiment. For example, a gene that is only regulated under very specific circumstances is unlikely to be modulated in many data sets that are curated in the knowledgebase. Thus, only a few entities that causally regulate the gene will exist in the knowledgebase. In contrast, a gene whose expression is modulated by a large number of experimental perturbations is likely to be modulated in many data sets that are represented in the knowledgebase, and thus the knowledgebase will likely contain knowledge of many entities that causally regulate the gene. Therefore, a comparable HYP is constructed by replacing each gene in the original HYP with another measured gene with a similar number of upstream controllers.

In order to accomplish this, all measured genes were ranked based on their number of upstream controllers in the knowledgebase, and divided into cadres of a fixed number of measurables. To avoid having a cadre containing only a few measurables (for example, when the number of measurables is not divisible by the desired cadre size), the cadre that contained measurables with the fewest number of upstream controllers was allowed to have more measurables than the other cadres. For example, a cadre size of 100 measurables was used, so the cadre that contained measurables with the fewest number of upstream controllers had between 100 and 199 members. Comparable HYPs were constructed by swapping each measurable in the original HYP for another measurable from within the same cadre. In this manner, the comparable HYPs were the same size as the original HYP and contained the same distribution of frequently and infrequently modulated measurables. This process enabled the construction of alternative HYPs that were as comparable as possible to the HYP of interest, given the knowledge available in the knowledgebase.

NPA scores were computed for each comparable HYP, and these scores were sorted into ascending order. The fraction of scores that were greater than and less than the score for the original HYP were counted, and the lesser of these two values was doubled to arrive at a two-tailed *p*-value. This *p*-value is called the Specificity.

Comparable HYPs constructed in this manner provide a more stringent test to assess the Specificity of amplitude scores. Given the benefits offered by this method of computing Specificity, it was carried forward for this study.

## Endnotes

^a^ The gene expression data used in this publication have been deposited in ArrayExpress and are accessible through accession number E-MTAB-1027 [37].

## Competing interests

The authors declare that they have no competing financial or other interests in relation to the work described in this manuscript.

## Authors’ contributions

FM, TMT, AS, and DAD lead the development, implementation, and application of the methodology, the interpretation of the results, and the preparation of the manuscript. CM and DW designed and supervised the experiment, and interpreted the experimental results. DP conceived the NPA approach and developed the methodology. JH developed the overarching strategy and supervised the project. MCP conceived the NPA approach and developed the overarching strategy. All authors read and approved the final manuscript.

## Supplementary Material

Additional file 1 **The NF-κB-direct HYP.** Each row of the table contains a causal statement describing the connection between NF-κB and one of its direct target genes. Click here for file

Additional file 2 **TNFα dose-dependent induction of NF-κB nuclear translocation.(a)** Non-treated NHBE cells show a diffuse cytoplasmic staining of NF-κB (left) while after adding TNFα to the culture medium (right), the nucleus of stimulated cells is strongly labeled (magnification 10X/0.3); **(b)** NF-κB nuclear fluorescence intensity (FI) per dose of TNFα. For each group, the nuclear fluorescence intensity was measured in 500 cells per well of three replicates. Across-group comparisons by one-way ANOVA test were all p < 0.001. Click here for file

Additional file 3 **HYP scores versus NF-κB nuclear translocation.** The NF-κB-direct HYP scores for each amplitude scoring method (Strength, GPI, EPI and MASS) and each time point (30 minutes, 2 hours, 4 hours, 24 hours) plotted against NF-κB nuclear translocation at 30 minutes. Score error bars represent the 95 % confidence interval as determined by the Uncertainty statistic. Error bars in NF-κB nuclear translocation represent the standard deviation of the mean nuclear translocation for three different fields of view of the same population of cells. Click here for file

Additional file 4 **Comparison of NF-κB-direct HYP scores with 20-gene NF-κB HYP scores.** Transcriptomic data from TNFα-treated NHBE cells was scored using each scoring method (Strength, GPI, EPI and MASS) for **(a)** the NF-κB-direct HYP, **(b)** a HYP composed of 20 NF-κB-regulated genes reported to be TNFα-responsive in mouse 3 T3 fibroblast cells (NFKBIA, CASP4, CCL5, TNFAIP3, CCL2, ZFP36, RIPK2, TNFSF10, NFKBIE, IL6, CCL20, ICAM1, TNFRSF1A, TNFRSF1B, SQSTM1, NRG1, SOD1, IL1RL1, HIF1A, ERBB2) [19]. Error bars represent the 95 % confidence interval as determined by the Uncertainty statistic. Scores that failed the Specificity criterion (Specificity *p*-value > 0.05; 1000 comparable HYPs) are shaded in gray. Click here for file

Additional file 5 **The aggregated IKK/NF-κB signaling HYP.** Each row of the table contains a causal statement extracted from the Selventa Knowledgebase and obtained from the aggregation of the individual HYPs of the IKK/NF-κB signaling causal network model (Additional file 6). Click here for file

Additional file 6 **The IKK/NF-κB signaling causal network model.** The full causal model is given (top), along with a schematic of the basic model architecture (middle). CHUK, IKBKB, and IKBKG act as inhibitors of NFKBIA, NFKBIB, and NFKBIE, which are in turn inhibitors of NFKB1, NFKB2, and RELA. The nodes used in the model are listed under each section. The nodes in bold represent nodes that have downstream gene expression measurables in the knowledgebase, and the number of measurables is given in the square brackets (because the same downstream may be found under multiple nodes, these 1227 downstream measurables correspond to 992 unique measurables). The notations used in the knowledgebase are as follows: “CHUK P@S” represents CHUK phosphorylated at serine (where the residue is given if known), “CHUK P@ST” represents CHUK phosphorylated at serine or threonine (the exact residue is unknown), “kaof(CHUK)” represents the kinase activity of CHUK, “CHUK:IKBKB” represents the complex of CHUK and IKBKB proteins, “IkappaB kinase complex Hs” represents an aggregate of the various IκB kinases (CHUK, IKBKB, and IKBKG) in Homo sapiens (Hs), “degradationof(NFKBIA)” represents the process of NFKBIA degradation, and “taof(NFKB1)” represents the transcriptional activity of NFKB1. Click here for file

Additional file 7 **The TNF HYP.** Each row of the table contains a causal statement extracted from the Selventa Knowledgebase and describes a gene known to be modulated by the TNFα treatment of cells. Click here for file

Additional file 8 **The E2F1-direct HYP.** Each row of the table contains a causal statement extracted from the Selventa Knowledgebase and describes the connection between E2F1 and one of its direct target genes. Click here for file

Additional file 9 **CellTiter-Glo® measurements of cell numbers.** CellTiter-Glo® fluorescence measurements of cell number after 24 hours of NHBE cell treatment with various doses of TNFα. Fluorescence intensity is reported as a percentage of the mock-treated sample, and error bars represent the standard deviation of the fluorescence intensity for three different fields of view of the same population of cells. Click here for file

Additional file 10 **Computing Specificity statistics.** The histogram of MASS scores for HYPs comparable to the NF-κB-direct HYP (1 ng/mL TNFα treatment of NHBE cells for 0.5 hour). The top histogram resulted from selecting measurables at random from all measurables (Specificity *p*-value of 0), and the bottom histogram resulted from selecting measurables with comparable likelihood of modulation (Specificity *p*-value of 0.072). The solid line indicates the NF-κB-direct HYP MASS score. The Specificity *p*-value was computed by doubling the total fraction of counts that were greater than this score (the fraction of counts in the red boxes). Click here for file
